# Older Adults’ Adoption of Mobility Devices: Interplay of Predisposing Factors and Precipitating Health Events

**DOI:** 10.1093/geroni/igaa057.1567

**Published:** 2020-12-16

**Authors:** Farhanaz Sharmin, Laura Sands

**Affiliations:** Virginia Tech, Blacksburg, Virginia, United States

## Abstract

Existing mobility limitations and chronic conditions increase likelihood of adopting mobility-related devices such as canes and walkers. Prior research has not considered how recent acute events such as falls and hospitalizations contribute to the adoption of mobility devices. We studied 4,592 older adults who responded to the 2015 and 2016 National Health and Aging Trends Study surveys, and classified adoption of mobility devices as: (i) Never users (did not use mobility devices either year) and (ii) New users (started using mobility devices in 2016). We determined through chi-square tests, that predisposing characteristics from 2015 that were significantly associated with being a New User in 2016 were: being female, aged 80+, minority race, having a high-school education or lower, living alone, being obese, and having a history of dementia, arthritis, stroke, mobility difficulties, falls, and hospitalization (all P’s<0.05). We used logistic regression to determine the contribution of recent precipitating events on the adoption of mobility devices among older adults after controlling for 2015 characteristics that were significantly associated with being a New user. Precipitating events were significantly associated with being a New user of mobility equipment. Specifically, older adults who, between the 2015 and 2016 interviews, experienced a fall (OR=1.7; 95% CI=1.1-2.9), hospitalization (OR=3.7; 95% CI=2.3-5.9) or increase in mobility difficulties (OR=3.7; 95% CI=2.3-5.9) were more likely to be New users. Study findings reveal the importance precipitating events on the adoption of mobility devices, signaling the importance of assessing for need for mobility devices after these events.

